# Liquid biopsy epigenetics: establishing a molecular profile based on cell‐free DNA

**DOI:** 10.1002/1878-0261.70145

**Published:** 2025-11-21

**Authors:** Christoffer Trier Maansson, Anders Lade Nielsen, Boe Sandahl Sorensen

**Affiliations:** ^1^ Department of Clinical Biochemistry Aarhus University Hospital Denmark; ^2^ Department of Clinical Medicine Aarhus University Denmark; ^3^ Department of Biomedicine Aarhus University Denmark

**Keywords:** cell‐free ChIP, cell‐free DNA, epigenetics, fragmentomics, liquid biopsy, methylation

## Abstract

Liquid biopsies containing circulating tumor DNA (ctDNA) are important biomarkers across several forms of cancer. The detection of mutations in cell‐free DNA (cfDNA) indicates the presence of ctDNA. However, unsatisfactory ctDNA mutation sensitivities, issues with sequencing errors, and clonal hematopoiesis variants have limited the clinical utility of mutation‐based ctDNA assays. Recently, a new avenue of cfDNA assays has been developed, focusing on cfDNA epigenetics. Here, we outline the recent advancements in cfDNA epigenetics, focusing on cfDNA methylation, fragmentomics, and post‐translational modifications (PTMs) of circulating nucleosomes. We present various methylation strategies concerning ctDNA detection and tissue of origin (TOO) analyses. cfDNA fragmentomics focuses on cfDNA fragment lengths, fragment end motifs, and nucleosome positioning to infer gene expression and estimate the ctDNA fraction. Lastly, we discuss the development of cell‐free chromatin immunoprecipitation of circulating nucleosomes with PTMs. This method has been implemented to detect tumor gene expression, TOO, and treatment resistance. Combining the epigenetic features of cfDNA will expand the utility of liquid biopsies to give a more comprehensive insight into tumor biology, treatment response, and resistance.

## Graphical abstract

Cell‐free DNA (cfDNA) fragments in plasma from cancer patients carry epigenetic signatures reflecting their cells of origin. These epigenetic features include DNA methylation, nucleosome modifications, and variations in fragmentation. This review describes the biological properties of each feature and explores optimal strategies for harnessing cfDNA epigenetic signals to advance cancer biology understanding and improve patient outcomes.

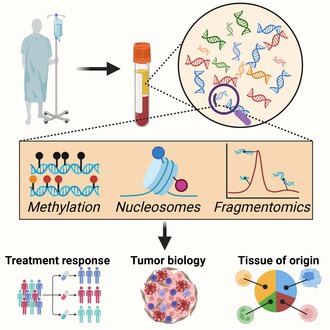

**Abbreviations**
5hmC: 5‐hydroxymethylcytosine
5mC: 5‐methylcytosine
AMI: acute myocardial infarction
AUC: area under the curve
CAD: caspase‐activated DNase
cfChIP: cell‐free chromatin immunoprecipitation
cfDNA: cell‐free DNA
cfMeDIP‐seq: cell‐free methylated DNA immunoprecipitation and high‐throughput sequencing
cfRNA: cell‐free RNA
CH: clonal hematopoiesis
ChIP: chromatin immunoprecipitation
citH3: citrullinated histone 3
CNA: copy number alteration
CRC: colorectal cancer
CTC: circulating tumor cell
ctDNA: circulating tumor DNA
CUP: cancer of unknown primary
ddPCR: droplet digital PCR
DMR: differentially methylated region
eccDNA: extrachromosomal circular DNA
EM‐seq: enzymatic methyl sequencing
EPINUC: epigenetics of plasma‐isolated nucleosomes
EV: extracellular vesicle
FEM: fragment end motif
H3K27ac: histone 3 lysine 27 acetylation
H3K36me3: histone 3 lysine 36 trimethylation
H3K4me3: histone 3 lysine 4 trimethylation
HCC: hepatocellular carcinoma
indel: insertion and deletion
lncRNA: long noncoding RNA
LUAD: lung adenocarcinoma
LUSC: lung squamous cell carcinoma
MBD2: methyl‐binding domain 2 protein
miRNA: micro‐RNA
ML: machine learning
mRNA: messenger RNA
NET: neutrophil extracellular trap
NGS: next‐generation sequencing
NSCLC: non‐small cell lung cancer
PBMC: peripheral blood mononuclear cell
PTM: post‐translational modification
qPCR: quantitative PCR
RCC: renal cell carcinoma
SCLC: small cell lung cancer
SNV: single‐nucleotide variant
TOO: tissue of origin
TSS: transcription start site
TTS: transcription termination site
WGBS: whole‐genome bisulphite sequencing
WGS: whole‐genome sequencing

## 1 Background

Liquid biopsies allow for longitudinal monitoring of tumor‐derived molecular alterations across multiple analytes in serial samples. Biological properties of cancers have been studied in the blood since the 19th century [1, 2], but the focus on liquid biopsy in cancer research has grown exponentially since the dawn of the 21st century alongside the development of next‐generation omics. Blood is the conventional biological sample used for liquid biopsies, but many other fluids, such as urine [3], cerebrospinal fluid [4], bile [5], and breast milk [6], have also been studied.

Cell‐free DNA (cfDNA) is present in blood plasma and has been studied extensively in translational and clinical cancer studies. Throughout the years, the primary focus has been on the detection of cfDNA originating from cancer cells, which is classified as circulating tumor DNA (ctDNA). Somatic variants detected in cfDNA are classified as ctDNA. However, only a minuscule fraction of the ctDNA pool contains somatic variants, given that most ctDNA molecules are genetically identical to cfDNA fragments from normal cells. This has led to the development of nonmutation‐based ctDNA detection assays, based on ctDNA methylation, ctDNA fragmentomics, and post‐translational modification (PTM) patterns of circulating nucleosomes.

## 2 Liquid biopsy analytes

**Fig. 1:**
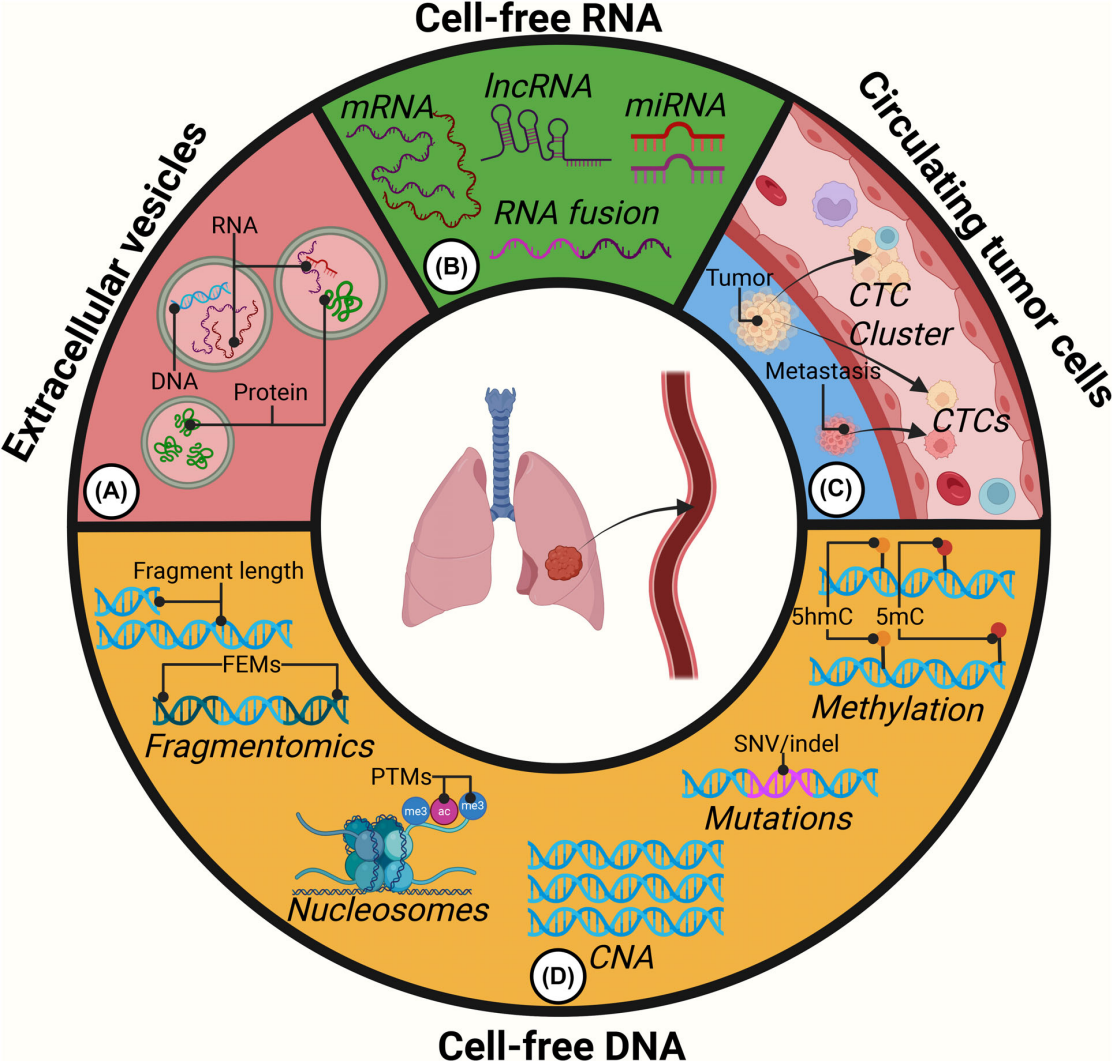
Various analytes of liquid biopsies. Cancer cells shed various analytes into the bloodstream, all of which can be analyzed in liquid biopsies. (A) Extracellular vesicles contain various molecules, including DNA, RNA, and proteins representing the biology of the tumor. (B) Several types of cell‐free RNA (cfRNA) molecules can be found in plasma, including noncoding micro‐RNA (miRNA), long noncoding RNA (lncRNA), messenger RNA (mRNA), and tumor‐derived RNA fusions. (C) Circulating tumor cells (CTCs) from primary tumors or metastatic sites directly represent cancer biology at the site of origin. CTCs can group together with healthy cells, creating CTC clusters. (D) Many features of cell‐free DNA (cfDNA) provide great insights into tumor biology. These features include fragmentomics focusing on cfDNA fragment lengths and fragment end motifs (FEMs), 5‐hydroxymethylcytosine (5hmC) and 5‐methylcytosine (5mC) patterns and post‐translational modifications (PTMs) of circulating histones. In addition, somatic variants, such as single‐nucleotide variants (SNVs), insertions and deletions (indels), and copy number alterations (CNAs) are detectable in liquid biopsies.

Many biomarkers can be quantified and studied in liquid biopsies, each of which has its own strengths and limitations (Fig. 1). The analytes include cell‐free RNA (cfRNA), extracellular vesicles (EVs), circulating tumor cells (CTCs), and cfDNA. In addition, circulating proteins, such as CEA and PSA, serve as important biomarkers across multiple cancer types [7, 8]. However, liquid biopsies traditionally refer to nucleic acid biomarkers [9]; therefore, we will not discuss circulating proteins further in this review.

### 2.1 Extracellular vesicles

Cancer‐derived EVs are not just a by‐product of cell death but are involved in cell‐to‐cell communication between, for example, tumor cells and the tumor microenvironment [10]. For example, small EVs can contain tumor‐derived mRNA providing valuable information about ovarian cancer patients [11], whereas other analyses focus on tumor genomics derived from EVs [12] (Fig. 1A). EVs can be isolated using surface markers, including EGFR and PD‐L1, to enrich tumor‐derived EVs [13] or using size exclusion chromatography, which catches a broader range of EVs [14].

### 2.2 Cell‐free RNA

Multiple types of cfRNA molecules have been found in plasma, including noncoding micro‐RNA (miRNA), long noncoding RNA (lncRNA), and coding messenger RNA (mRNA) (Fig. 1B). Some researchers have focused on identifying gene expression in the tissue of origin (TOO) by performing whole‐transcriptome sequencing of cfRNA [15, 16, 17], whereas others have focused on the quantification of tumor‐specific cfRNA targets [18, 19, 20, 21, 22]. For example, six specific cfRNA candidates were systematically shown to be enriched in hepatocellular carcinoma (HCC) and could be used to distinguish healthy individuals from HCC patients in an independent cohort [19]. Focusing on mutations, Hasegawa et al. [18] identified the most recurrent gene fusions in lung cancer involving *ALK*, *RET*, and *ROS1* in 8/30 (27%) of the cfRNA samples examined from non‐small cell lung cancer (NSCLC) patients. Although cfRNA studies are promising, the optimal preanalytical methodology for cfRNA isolation and processing remains unresolved [23], creating challenges in cross‐study comparisons [24].

### 2.3 Circulating tumor cells

CTCs come directly from the tumor tissue and therefore represent the properties of the primary tumor and metastatic sites (Fig. 1C). For example, CTCs carry tumor mutations [25] and RNA profiling of CTCs can be used to predict treatment resistance to targeted therapies [26] or identify distinct tumor cell phenotypes [27]. Moreover, CTCs and CTC clusters of tumor and immune cells can give rise to metastases and therefore serve as cancer biomarkers [28, 29, 30]. CTCs are vastly outnumbered in the circulation, leading to several strategies for isolating CTCs, including technologies focusing on surface markers [31] and marker‐independent microfluidic devices [32].

## 3 Cell‐free DNA

cfDNA is extracellular DNA that is constitutively present in plasma in all individuals (Fig. 1D). Various pathological conditions can increase the level of cfDNA, including cancer [33], infections [34], and tissue trauma [35]. In addition, multiple nonpathological situations, such as pregnancy and exercise, lead to increased cfDNA content [36].

### 3.1 Origin and release of cfDNA

cfDNA is cellular DNA released into circulation after cell death, either by apoptosis or necrosis [37]. Apoptosis leads to short cfDNA fragments (< 400 bp) with a fragment size distribution reflecting mono‐ and di‐nucleosomes, whereas necrosis is the spontaneous release of long cfDNA fragments. Apoptosis is considered the primary source of cfDNA, given that the fragmentation of cfDNA is nonrandom, meaning it is dependent on the underlying chromatin structure [38]. Where apoptosis and necrosis result in passive cfDNA release mechanisms, it is now also recognized that cfDNA can be actively released into the bloodstream. One example of active release is the development of neutrophil extracellular traps (NETs) [39], which are disintegrated chromatin that can trap microorganisms [40]. Exercise has been shown to increase the cfDNA concentration [41], which is predominantly caused by an increase in neutrophil cfDNA [42, 43]. Beiter et al. [43] observed an increase in NET‐like structures following exercise. In support of this, Fridlich et al. [42] applied H3K4me3 cell‐free chromatin immunoprecipitation sequencing (cfChIP‐seq) to evaluate the cfDNA tissue of origin following exercise and observed a large increase in the neutrophil fraction. Importantly, exercise also increased the level of citrullinated histone 3 (citH3), which is a hallmark of NETs, and citH3 levels correlated with neutrophil cfDNA contents [42]. These results demonstrate that the increased cfDNA concentration observed during or after exercise is at least in part caused by active release of NETs.

**Fig. 2:**
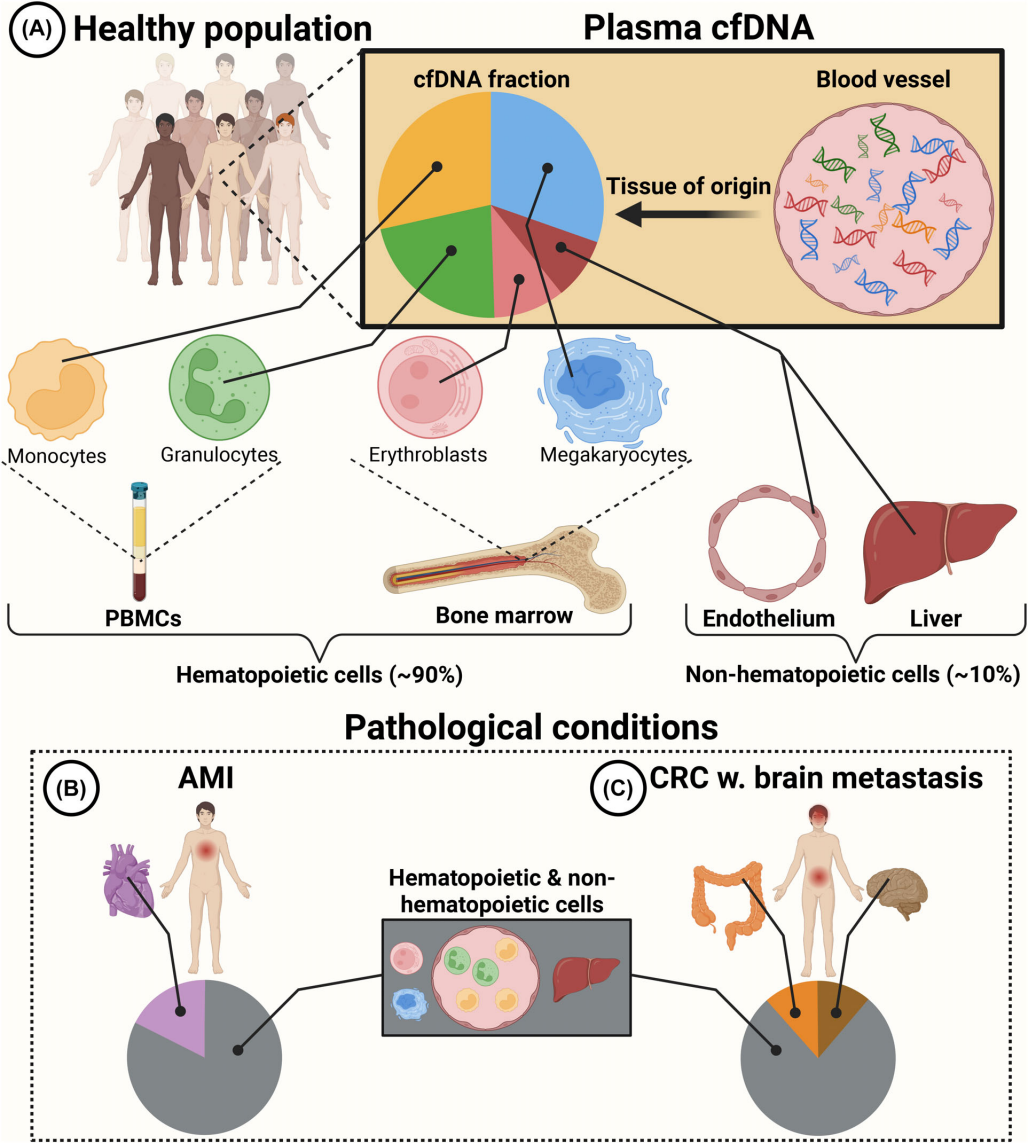
Origin of cell‐free DNA (cfDNA) in healthy individuals and various diseases. (A) In a healthy population, the tissue of origin of cfDNA can be traced to hematopoietic (90%) and nonhematopoietic cells (10%). Of circulating peripheral blood mononuclear cells (PBMCs), monocytes (yellow) and granulocytes (green) are the main contributors to the cfDNA pool. Erythroblasts (pink) and megakaryocytes (blue) are the main contributors from the bone marrow. Nonhematopoietic cfDNA primarily originates from endothelial and liver cells (red). (B, C) Pathological conditions can alter the origin of cfDNA; however, most of the cfDNA still originates from healthy tissues shown in (A) (gray). Patients with acute myocardial infarction (AMI) have increased cfDNA from cardiac (purple) cells (B). Similarly, cancer patients have an increased fraction of cfDNA from the organ of the primary tumor and the metastatic site (C). This is illustrated with an increased fraction of colon (orange) and neuron (brown) cfDNA in a colorectal cancer (CRC) patient with a brain metastasis.

Another active release mechanism is the enucleation by erythroblasts during the formation of erythrocytes in the bone marrow [44]. This aligns with two initial cfDNA methylation studies from Lam et al. [45] and Moss et al. [46] estimating that about 30% of the cfDNA originates from erythrocyte progenitors in healthy individuals. However, this estimate has been markedly reduced to approximately 5% in subsequent cfDNA methylome studies [47, 48, 49]. The initial overestimation of erythroblast cfDNA was likely caused by incomplete reference methylomes that Lam et al. and Moss et al. used. They did not consider megakaryocyte contribution to the plasma cfDNA pool, which were later shown to account for approximately 30–45% of cfDNA in healthy individuals [47, 48]. Outside of cfDNA methylomes, multiple approaches, such as cfDNA fragmentomes [50] and epigenetic marks on circulating nucleosomes [35], have been used to estimate the relative contribution of cfDNA from various tissues. The overall consensus is that more than 90% of cfDNA in healthy individuals originates from hematopoietic cells (Fig. 2A) [35, 47, 50, 51, 52]. Researchers exploring the cfDNA cell of origin have identified granulocytes, megakaryocytes, erythrocyte progenitors, and monocytes/macrophages as the main contributors to the hematopoietic‐derived cfDNA [35, 46, 47]. Nonhematopoietic cfDNA in healthy individuals, representing about 10%, mainly originates from endothelial and hepatic cells [35, 46, 47].

The origin of cfDNA is altered in various pathological conditions. The anatomical site of the disease increases the cfDNA contribution from the adjacent tissue by collateral damage to the healthy cells at that site [53]. Examples include the increase in megakaryocyte cfDNA in thrombocythemia patients [49], heart cfDNA in acute myocardial infarction patients (Fig. 2B) [35, 54], and neuron or liver cfDNA in cancer patients with metastases in the brain or liver, respectively (Fig. 2C) [53].

### 3.2 Clearance of cfDNA

The cfDNA levels depend on the amount of cfDNA released and the level of cfDNA clearance. The exact half‐life of cfDNA is still debated; however, most studies estimate the half‐life from a few minutes to 1–2 h [55, 56, 57, 58]. The inconsistent estimate of the cfDNA half‐life is likely caused by the differences between cfDNA quantification techniques and study populations, including postpartum women [56], postsurgery cancer patients [57], healthy individuals postexercise [58], and patients following hemodialysis [55]. Given that cfDNA degradation is complex, involving the liver, kidneys, and spleen, the functions of these organs have likely varied between the individual studies. The best estimate of the mononucleosomal cfDNA (100–250 bp) half‐life in healthy individuals is probably 24.2 min (range: 14.9–32.7 min, *n* = 5), reported by Yamamoto et al. [58]. In this study, the authors reduced variance by accounting for fragment sizes, inhibited postsampling cfDNA degradation, quantified cfDNA without PCR, and used healthy individuals as the study population. However, a larger cohort with a more diverse human population is needed to validate this estimate.

cfDNA is primarily degraded through macrophages in the liver, which can uptake mononucleosomes and digest the cfDNA [59, 60]. cfDNA fragments can also be directly excreted through the kidneys, where the urine cfDNA fragment length distribution is markedly different from that of plasma cfDNA [61]. Urine cfDNA is shorter and lacks the characteristic ~ 165‐bp peak fragment size observed in plasma [61, 62]. Instead, urine cfDNA has a modal size of approximately 81 bp with sharp 10‐bp periodicity, which reflects the enzymatic degradation of urine cfDNA [61]. The difference between plasma and urine fragment size profiles could indicate that short plasma cfDNA fragments unprotected from histone proteins are more rapidly excreted to the urine. However, proteomic studies have revealed histone proteins in the urine, which indicates that protein‐bound cfDNA also can be filtered from plasma to urine [63] and the proteins can transiently protect the cfDNA from degradation [61].

cfDNA can also be degraded in the circulation by circulating DNases, such as DNASE1, DFFB, and DNASE1L3 [64], where DNASE1L3 is likely the primary DNase responsible for the degradation of cfDNA in plasma [65]. Several factors can affect the effect of these nucleases on cfDNA degradation, including the prandial state [66]; the level of anti‐DNA antibodies [59]; and various pathological conditions, such as cancer [67]. Malki et al. [68] demonstrated that healthy individuals with high concentrations of cfDNA had decreased DFFB and DNASE1L3 activity. In accordance, in a genome‐wide association study, Linthorst et al. [69] identified a common variant in *DNASE1L3* (p. Arg206Cys) linked to altered cfDNA profiles. This variant was observed in 7% of a Dutch population and resulted in significantly reduced cfDNA concentrations and altered fragmentation profiles with an increased frequency of very short cfDNA fragments (< 100 bp). Additionally, the cfDNA concentration has been shown to negatively correlate with the fraction of short fragments (< 160 bp) [68]. Correspondingly, the cfDNA concentration positively correlated with the fraction of long fragments (> 231 bp). Collectively, these results illustrate how increased DNase activities, particularly DNASE1L3, lead to lower cfDNA concentrations and a larger fraction of short cfDNA fragments [68].

### 3.3 Somatic mutation detection

In 1994, Sorenson et al. and Vasioukhin et al. described how *RAS* mutations detected in the tumor could also be detected in the blood [70, 71]. This has led to countless studies evaluating ctDNA in cancer patients before, during, and after treatment [72, 73]. Several types of somatic variants can be detected in ctDNA, including single‐nucleotide variants (SNVs) [70, 71], insertions and deletions (indels) [74], gene fusions [75], and copy number alterations (CNAs) [76] (Fig. 1D). It has often been stated that ctDNA levels increase with advancing cancer stages and that different types of cancer shed different levels of ctDNA [77]. Two applications of ctDNA monitoring have been studied most extensively: (a) the detection of minimal residual disease after treatment with curative intent [78, 79, 80, 81] and (b) the evaluation of treatment responses in advanced cancer [74, 82, 83, 84, 85]. The detection of somatic mutations in ctDNA, unique to the tumor cells, is a strong indication of residual disease following treatment with curative intent [57]. Given that the half‐life of cfDNA is short, tumor dynamics can be monitored in real time through a minimally invasive procedure [56]. ctDNA dynamics during treatment of cancer patients can also be used to stratify cancer patients as molecular responders and nonresponders, which reflect the clinical response [86].

ctDNA detection methods vary between individual studies, where some focus on tumor‐naïve approaches using, for example, commercially available next‐generation sequencing (NGS) platforms, and others use tumor‐informed strategies with, for example, NGS or droplet digital PCR (ddPCR). The various ctDNA detection methodologies have distinct strengths and weaknesses in terms of ctDNA sensitivity, turn‐around time, cost, and flexibility, which have been described in great detail [86, 87, 88, 89]. However, in any mutation‐based ctDNA detection study, the genetic consequences of clonal hematopoiesis (CH) have to be taken into consideration [90]. CH describes the selective propagation of clonal populations of blood cells, and cfDNA variants from these cells can be misinterpreted as tumor‐derived ctDNA mutations [91]. An approach to overcome this limitation is to focus on ctDNA epigenetics, which does not rely on the identification of somatic variants.

## 4 cfDNA epigenetics

The epigenetic profile of cfDNA represents the molecular landscape in the cells of origin. Even though cfDNA represents a pool of DNA from multiple cell types, multiple epigenetic properties of the cfDNA can be utilized to detect ctDNA or understand molecular pathways active in specific tissues. These strategies can increase cfDNA's utility by providing a more comprehensive molecular profile of the tumor cells reflecting, for example, transcriptional programs, molecular/histological subtypes, and tumor cell plasticity.

### 4.1 cfDNA methylation

**Fig. 3:**
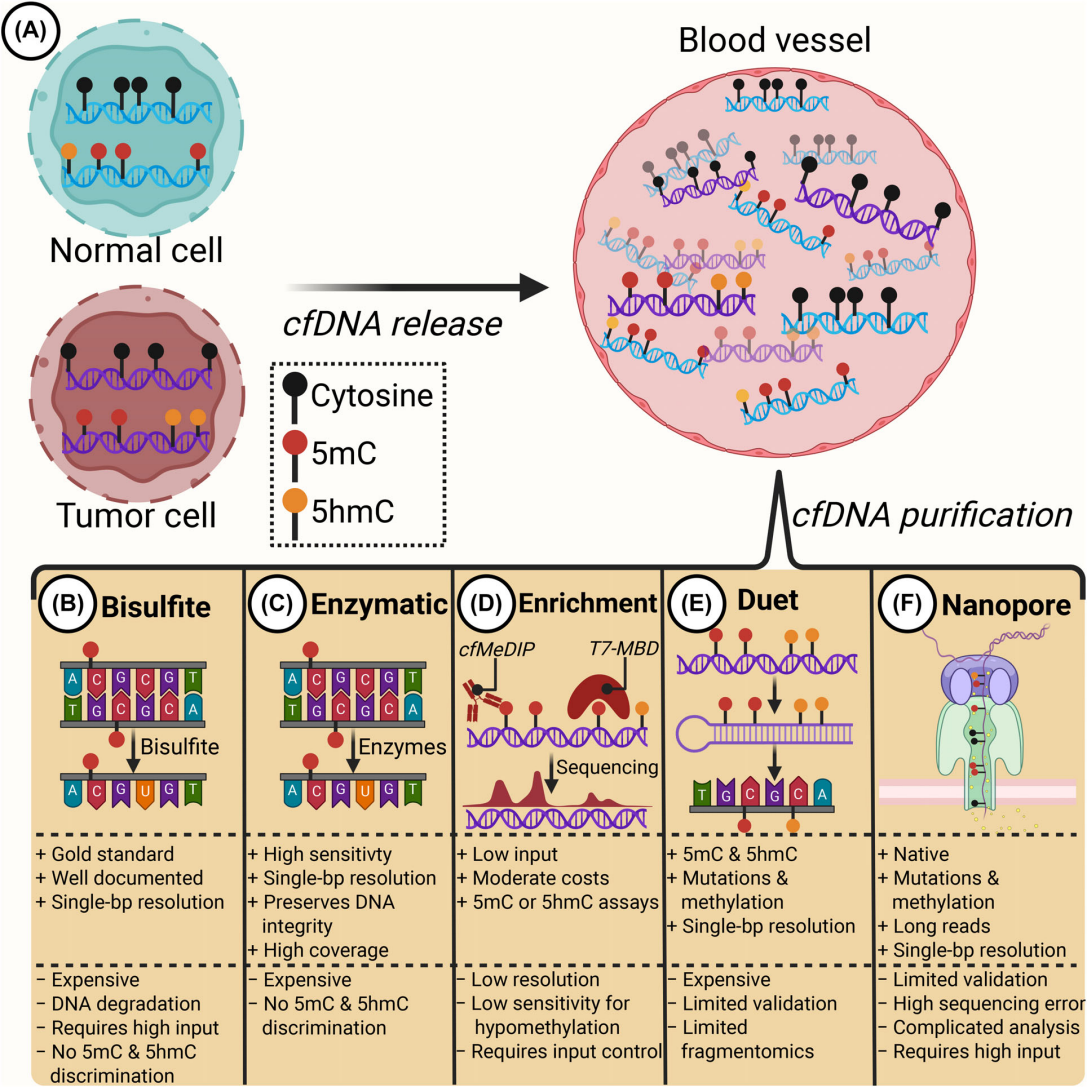
Different cell‐free DNA (cfDNA) methylation detection methods. (A) Normal and tumor cells have different methylation profiles, which are detectable in the bloodstream. Several strategies for cfDNA methylation quantification exist, each with their own strengths (+) and limitations (−). (B) Bisulfite conversion is the gold standard of methylation profiling; however, it is limited by high DNA degradation requiring a high input content. (C) As an alternative to bisulfite, enzymatic methyl sequencing (EM‐seq) preserves the DNA integrity while providing single base‐pair (bp) resolution of 5‐methylcytosine (5mC) with higher coverage and less GC bias than bisulfite conversion. (D) Two separate 5mC cfDNA enrichment strategies exist, cell‐free methylated DNA immunoprecipitation and high‐throughput sequencing (cfMeDIP–seq) and T7‐MBD‐seq. cfMeDIP utilizes anti‐5mC‐DNA antibodies to isolate methylated cfDNA fragments, whereas T7‐MBD‐seq uses methyl‐binding domain 2 protein (MBD2) to enrich for 5mC. Precipitated cfDNA is sequenced, generating a coverage profile representing the genomic region's methylation status. (E) 6‐letter sequencing with Duet is one of the more recently developed methods and enables quantification of 5mC and 5‐hydroxymethylcytosine (5hmC) at single‐bp resolution. (F) Native cfDNA is sequenced with long reads using nanopore technology, generating both an epigenetic and genetic profile at single‐bp resolution.

5‐Methylcytosine (5mC) of CpG sites is a common epigenetic feature of the genome [92]. Differentially methylated regions (DMRs) can be discovered in liquid biopsies, and because different cell types have different methylation patterns, the cell of origin can be determined by analyzing genome‐wide 5mC patterns of cfDNA [46, 47, 53, 93] (Fig. 3A).

#### 4.1.1 Methods to quantify cfDNA methylation

Traditionally, 5mC cfDNA has been detected by performing bisulfite conversion and thereby identifying specific loci with 5mC (Fig. 3B). For example, Lehmann‐Werman et al. [94] used this technique to identify the cfDNA contribution from β‐cells by focusing on methylation in the insulin promoter in type 1 diabetic patients. Moreover, Sun et al. [52] used whole‐genome bisulfite sequencing (WGBS) to estimate the cfDNA origin and showed that patients with HCC had an increased liver cfDNA fraction. However, a disadvantage of bisulfite‐based 5mC detection is the cfDNA degradation caused by the bisulfite conversion and that 5‐hydroxymethylcytosine (5hmC) cannot be discriminated from 5mC. As an alternative to WGBS, enzymatic methyl sequencing (EM‐seq) assays are based on the enzymatic conversion of 5mC and 5hmC, followed by sequencing [95] (Fig. 3C). The DNA integrity is better preserved with EM‐seq, resulting in higher coverage, lower duplicate rates, and less GC content bias than with WGBS [96, 97].

Another approach has been to use cfDNA methylation enrichment strategies, such as cell‐free methylated DNA immunoprecipitation and high‐throughput sequencing (cfMeDIP–seq) to identify DMRs [98] (Fig. 3D). This technique uses anti‐5mC antibodies to isolate cfDNA with 5mC, creating a genome‐wide enrichment profile representing the methylation patterns of the TOOs. Another 5mC enrichment strategy is T7‐MBD‐seq, which utilizes methyl‐binding domain 2 protein (MBD2) to enrich methylated cfDNA [99]. Interestingly, this technique has recently been used to classify the location of the primary tumor in cancer of unknown primary (CUP) patients and therefore serves as a potential diagnostic tool for these patients [93]. Although the performance and output of T7‐MBD‐seq and cfMeDIP‐seq are similar, T7‐MBD‐seq has been shown to produce better data quality and a lower duplicate rate [100]. A limitation of both methods is that isolating 5mC cfDNA using antibodies or MBD2 only estimates the methylation on a fragment level and therefore cannot quantify the methylation for each CpG site (β‐values) in the analyzed cfDNA fragment. Moreover, it is more difficult to identify hypomethylated regions using these techniques. In a study by Loyfer et al. [47] investigating methylomes in 39 healthy cell types with WGBS, 97% of the DMRs specific for different cell types were hypomethylated. These regions were close to genes reflecting the cell phenotype and function and were enriched for markers of active transcription, including histone 3 lysine 27 acetylation (H3K27ac). In theory, these cell‐specific hypomethylated regions would be difficult to quantify using enrichment‐based 5mC cfDNA protocols.

More recent methods include combined genomic and methylation analyses, such as 5/6‐letter (Duet) sequencing [101] (Fig. 3E) and native DNA sequencing with nanopore [102] (Fig. 3F), which have expanded the cfDNA methylation field. The advantage of 6‐letter sequencing is the ability to quantify 5mC and 5hmC while also providing a complete genetic sequence for the detection of mutations [101]. A disadvantage of the Duet technology, however, is that cfDNA fragment size estimations are limited by the read length, meaning at least 250‐base paired‐end sequencing is needed to capture some fragmentomic features of cfDNA. This is not a limitation with nanopore sequencing, where long reads are used to establish a native epigenetic and genetic cfDNA profile [102]. Although the data are still limited, promising studies have shown that cfDNA methylome and fragmentome differences between healthy individuals and cancer patients can be detected with the nanopore technology [102, 103]. The sequencing error rate with nanopore remains a limitation; however, the accuracy has increased through the years, improving its utility [104]. In summary, both Duet and nanopore technologies are still in their infancy and require further validation and optimization before application in clinical trials.

#### 4.1.2 Application of cfDNA methylation

Regardless of the method, cfDNA methylomics has been used extensively to study cancer diseases. This is seen in the case of lung cancer, where some studies focus on a small number of DMRs specific to the cancer type of interest [105, 106]. These targets are easily identifiable using bisulfite conversion followed by quantitative PCR (qPCR) specific to the unmethylated and methylated alleles [107]. Wang et al. [105] developed two models containing different CpG marks for lung cancer screening and lung cancer diagnosis. The screening model achieved an area under the curve (AUC) of 0.90 (95% CI = 0.85–0.94) when comparing healthy individuals to cancer patients. The diagnostic model, on the other hand, achieved an AUC of 0.81 (95% CI = 0.78–0.86) when comparing patients suffering from benign lung diseases with lung cancer patients. Other studies focus on even fewer targets, such as *SHOX2* and *PTGER4* [106]. Here, Weiss et al. developed a qPCR‐based assay to detect differentially methylated sites and obtained an AUC of 0.86 when comparing lung cancer patients to nonmalignant lung diseases. 5mC‐qPCR offers an advantage over somatic mutation analyses by providing a universally applicable assay regardless of tumor‐specific mutations. This approach proves highly cost‐efficient and straightforward to implement. However, the method presents challenges in marker selection, as the methylation sites must be exclusively tumor‐specific, avoiding their presence in normal adjacent tissue or hematopoietic cells contributing to the cfDNA pool. Furthermore, cancer cells frequently alter the genome‐wide distribution of 5mC [108], complicating the identification of consistent and stable methylation sites.

A potential solution to circumvent the dynamics of 5mC and to increase the sensitivity and specificity of methylation‐based ctDNA detection is to develop a model based on thousands of targets. This has resulted in the development of genome‐wide cfDNA methylation approaches across multiple cancer types [46, 47, 53, 93, 99]. Moss et al. developed a human cell‐type methylation atlas [46], which the same group later expanded [47]. Although these studies primarily focus on methylation patterns in normal cells, they have laid the groundwork for future cancer cfDNA methylation studies. Conway et al. [93] used T7‐MBD‐seq to create a machine learning (ML) classifier that can identify the TOO for cfDNA fragments and correctly classify the primary tumor in 23/26 (88.5%) CUP patients. Chemi et al. [99] focused on small cell lung cancer (SCLC), using the same enrichment‐based method. They developed a classifier that identified SCLC patients from healthy individuals, where extensive‐stage SCLC patients were more confidently identified (AUC = 1.000), compared with low‐stage SCLC patients (AUC = 0.986). In addition, they show how cfDNA methylome profiling can be used to identify SCLC subtypes, such as SCLC‐A and SCLC‐N, with multiple expression profiles. Shen et al. [98] reported similar results across multiple tumor types. They applied cfMeDIP‐seq and were able to differentiate among, for example, pancreatic cancer (AUC = 0.918), lung cancer (AUC = 0.971), and healthy individuals (AUC = 0.969). The performance of the classifier improved with advancing stages of cancer.

#### 4.1.3 Hydroxymethylcytosine

Most cfDNA methylation studies focus on 5mC, but more recently, 5hmC has also been evaluated as a potential biomarker in cancer [109, 110, 111]. 5hmC was previously thought to be an intermediate of 5mC demethylation [112] but is today established as a stable epigenetic mark [113, 114]. Li et al. [109] demonstrated how 5hmC correlated with markers of active transcription, and circulating 5hmC signatures could differentiate healthy individuals from gastric cancer (AUC = 0.95) and colorectal cancer patients (AUC = 0.93). Similarly, Cai et al. [110] demonstrated that circulating 5hmC in 32 marker genes could differentiate early‐HCC (AUC = 0.884) and late‐HCC patients (AUC = 0.905) from non‐HCC individuals. More recently, Sjöström et al. [115] demonstrated that cell‐free 5hmC signatures specific for prostate cancer correlated with the ctDNA content estimated with ichorCNA. Interestingly, high cell‐free 5hmC levels in the genes *EZH2* and *TOP2A*, where high expression is associated with aggressive prostate cancer, were observed in patients with short overall survival. These results show that 5hmC is closely related to gene transcription, and circulating 5hmC reflects the tumor's gene expression profile, highlighting cfDNA 5hmC as an important epigenetic biomarker.

### 4.2 cfDNA fragmentomics

cfDNA fragmentomics is the study of the nonrandom fragmentation of cfDNA caused by various biological mechanisms. cfDNA fragmentation caused by apoptosis is dependent on caspase‐3, which is activated by both the intrinsic and extrinsic apoptotic pathways. Caspase‐3 in turn activates the endonuclease, caspase‐activated DNase (CAD) [116]. CAD cleaves nuclear DNA in internucleosomal regions [117], which is reflected in the cfDNA size distribution [37]. Most cfDNA comprises approximately 165 bp, which corresponds to the 145 bp of DNA wrapped around the histone octamer in the nucleosome plus a 20‐bp linker region [37]. A smaller fraction of cfDNA has a size of approximately 340 bp, reflecting an underlying di‐nucleosome structure [118].

**Fig. 4:**
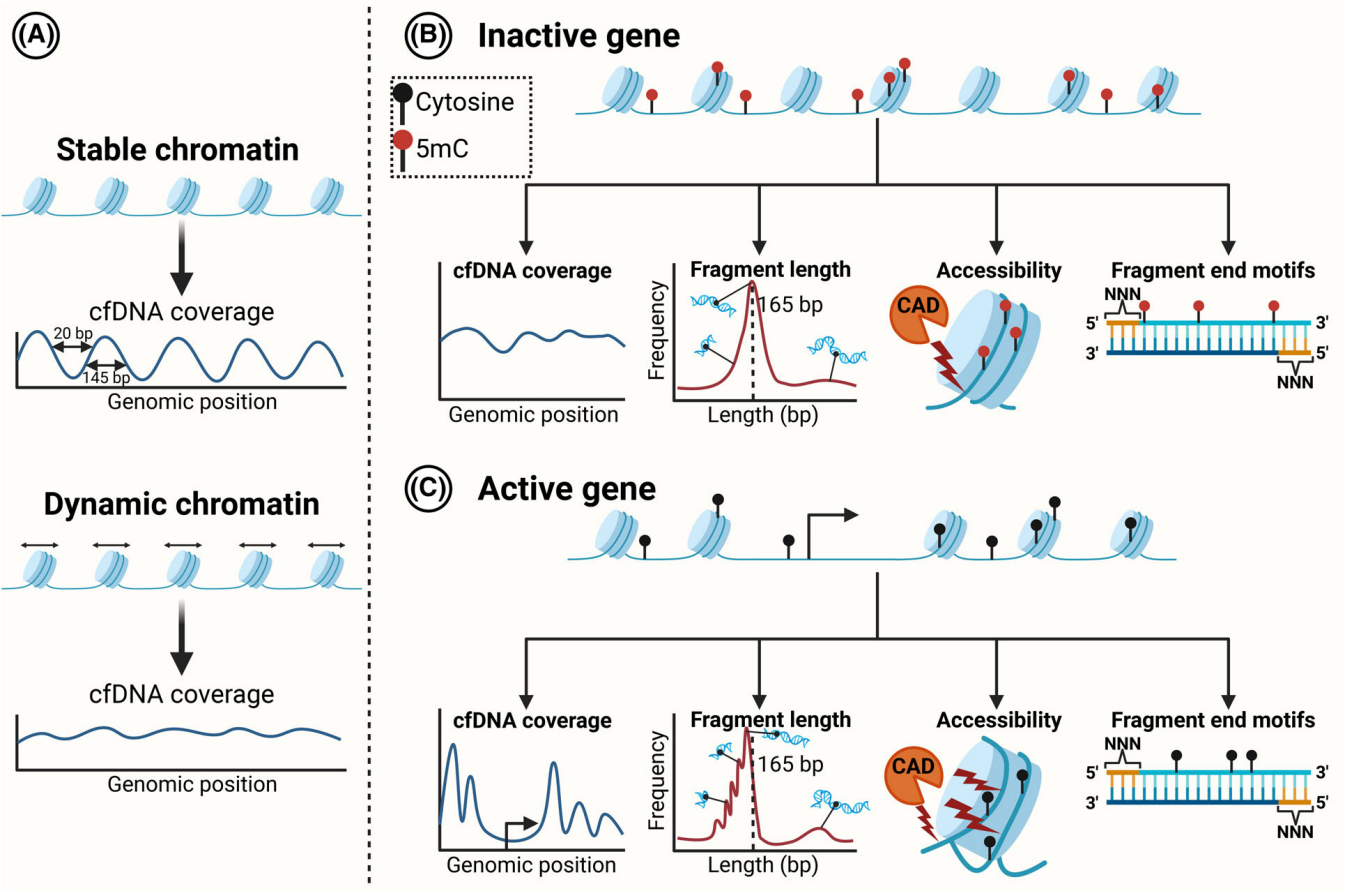
Fragmentomics of active and inactive genes. (A) Stable regions in the genome result in cell‐free DNA (cfDNA) coverage peaks and valleys corresponding to the underlying chromatin structure. Regions with more dynamic chromatin result in a flatter cfDNA coverage profile. (B) Inactive genes are enriched for 5‐methylcytosine (5mC) and do not have a structured cfDNA coverage profile around the transcript start site (TSS). cfDNA fragments from inactive genes have a peak at approximately 165 bp corresponding to caspase‐activated DNase (CAD) cleavage in internucleosomal regions, leading to distinct fragment end motifs (FEMs). (C) Active genes are depleted of nucleosomes around the TSS, leading to distinct cfDNA coverage profiles. cfDNA fragments from active genes are shorter, with approximately 10‐bp oscillations. Gene activity is also characterized by hypomethylation, causing the DNA to be more loosely bound to the nucleosome, allowing for internucleosomal CAD cleavage and altered FEM frequencies.

Because of the mechanism of degradation, the cfDNA fragmentation profile is dependent on the structure of the chromatin, and from the cfDNA coverage profiles, a nucleosome footprint can be inferred [50, 119, 120, 121, 122] (Fig. 4A). The cfDNA coverage profile refers to the variance in the cfDNA content across the genome, resulting in differences in sequencing depth. Genomic regions with structured chromatin across multiple cell types are represented with a distinct cfDNA coverage profile with peaks and valleys. Peaks represent regions with high relative content, whereas the valleys are regions depleted of cfDNA fragments. Other regions with dynamic chromatin have a flatter and more unstructured cfDNA coverage profile. Importantly, the chromatin structure is relatively fixed around active transcription start sites (TSSs), meaning gene expression can be inferred based on the coverage profiles around these regions (Fig. 4B,C) [50, 119, 120, 123]. cfDNA fragmentomics is typically investigated with whole‐genome sequencing (WGS), yielding a detailed genome‐wide fragmentomics profile of circulating DNA originating from multiple tissues [50, 122, 124, 125]. Using cfDNA WGS, nucleosome‐depleted TSS regions specific to individual tissues can be investigated to estimate the TOO across multiple pathological conditions [120]. For example, the estimated signals from liver and intestine cells have been shown to be upregulated in HCC and colorectal cancer (CRC) patients, respectively, compared with healthy subjects. Ulz et al. [126] also included transcription factor binding sites in their analyses and found that transcription factors could also protect the DNA from degradation. More recently, Esfahani et al. developed an NGS panel to specifically target promoter regions (called EPIC‐seq) [121]. Active genes have a more diverse cfDNA fragment length profile around the promoter (Fig. 4C), with more cfDNA fragments shorter than 150 bp and containing size peak oscillations approximately every 10 bp [118, 127]. This phenomenon reflects the consistent orientation of DNA wrapped around nucleosomes, where about 10.4 bp are required to complete a full DNA helix turn [50]. EPIC‐seq captures these features and was used to estimate a gene‐specific fragmentation entropy score, which correlated with gene expression in the cells of origin. EPIC‐seq could classify lung cancer patients from healthy individuals, with an AUC = 0.83 (95% CI = 0.71–0.96). In addition, EPIC‐seq was used to investigate gene expression differences between lung adenocarcinomas (LUADs) and squamous cell carcinomas (LUSCs), where the identification of LUAD reached an AUC = 0.90 (95% CI = 0.83–0.97). EPIC‐seq is different from most cfDNA fragmentomics studies because it relies on targeted sequencing of cfDNA rather than WGS. As a result, EPIC‐seq has high coverage in promoter regions, which allows gene expression to be estimated at the single‐gene level. WGS studies have lower sequencing depth, making single‐gene expression estimates more subject to technical variance but allowing fragmentomic features to be studied throughout the gene body [122].

Although some studies have focused on inferring expression and tissue contribution from cfDNA, other studies have focused on ctDNA detection based on fragmentomics [128]. This idea originates from the observation that fragments harboring ctDNA mutations are generally shorter than the corresponding WT cfDNA fragments from normal cells [127, 129]. Moreover, cancer patients have a different cfDNA size profile, caused by an increased fraction of short (< 150 bp) cfDNA fragments [118, 123]. Why cancer patients have an increased fraction of short fragments remains largely unknown. CAD cleaves genomic DNA during apoptosis; however, other endonucleases, such as DNASE1L3, have been shown to cooperate with CAD during apoptosis [130]. Interestingly, the inhibition of DNASE1L3 affects the cfDNA fragment length distribution [64]. It has been proposed that the nucleases DNASE1L3 and DFFB cleave the DNA within the cell whereas DNASE1L3 and DNASE1 further digest the DNA in the extracellular environment [131]. Studies with pregnant women have shown that the hypomethylated placental cfDNA is bound more loosely in the nucleosome [132], increasing accessibility for endonucleases, resulting in a different fragmentation profile than that of maternal cfDNA [133]. Similarly, cancer cells often exhibit genome‐wide DNA hypomethylation [108], which could explain why cfDNA is more degraded if hypomethylated DNA is less tightly associated with nucleosomes (Fig. 4B,C). These differential fragmentation ctDNA patterns have been utilized to develop ML classifiers to differentiate between cancer patients and healthy individuals. Cristiano et al. [125] established DELFI, which is an ML algorithm based on WGS of cfDNA that can detect ctDNA from multiple cancer types. For example, DELFI has been used to screen for lung cancer, resulting in a sensitivity of 0.84 (95% CI = 0.79–0.88) and specificity of 0.53 (95% CI = 0.45–0.61) in the validation dataset, which consists of 248 individuals with cancer and 134 individuals without cancer [134]. The ML classifier performed better in SCLC cases compared with NSCLC patients and had an increased sensitivity with advancing stages. DELFI scores in serial blood samples taken from CRC patients receiving curative‐intended treatment showed how DELFI dynamics can be used to identify patients with a risk of relapse [135]. Combined, these results illustrate how cfDNA fragmentomics can be applied in similar clinical contexts as mutation‐based ctDNA detection strategies. Although the results are promising, the involvement of ML classifiers might complicate the implementation in clinical practice, given external validation is vital to ensure the models are not overfitted to the training data [136]. A thorough presentation of ML applied on cfDNA, including some of the issues with these strategies, has been published elsewhere [137].

#### 4.2.1 cfDNA fragment end motifs

The study of fragment end motifs (FEMs) is another research area within cfDNA fragmentomics that has received attention over the last few years [123, 124]. FEMs are the nucleotide combinations present at the ends of cfDNA fragments. The number of base pairs in the FEMs varies between studies. A study on HCC focused on 4‐mers (4‐nucleotide sequences) and showed that distinct 4‐mers are associated with HCC and the activity of the endonuclease, DNASE1L3 [138]. Other studies have focused on 3‐mers. For example, Moldovan et al. [124] found that the combination of fragment size and FEM features in an ML model could distinguish cancer patients from healthy individuals (AUC = 0.96). Other approaches have focused on the frequency of single nucleotides at various positions relative to the fragment end, thereby not only evaluating nucleotides within the fragment but also the nucleotides flanking the cfDNA [139]. Combining the GC content, fragment lengths, and single‐nucleotide frequencies in a random‐forest ML model, it was possible to distinguish cancer patients from healthy individuals with an AUC = 0.91 [139].

A notable advancement has been the development of FRAGMA, a FEM‐based analytical approach that leverages the relationship between CpG‐FEM frequencies and their underlying methylation status [140]. Zhu et al. [141] were able to expand on FRAGMA to also consider nucleosomal fragmentation patterns, creating FRAGMAXR. Evaluating FRAGMAXR signals in differentially methylated regions between HCC patients and healthy individuals, they predicted the ctDNA content, which correlated with ichorCNA‐estimated ctDNA fractions (Spearman *r* = 0.96). In addition, a FRAGMAXR‐based ML classifier could differentiate HCC patients from healthy individuals with an AUC = 0.93. Revealing another use of FEMs, Wang et al. [142] developed an ML algorithm based on FEMs and MRI results to monitor chemotherapy treatment responses in local rectal cancer.

Although cfDNA FEMs have the potential to identify features distinguishing cancer patients from healthy individuals, the underlying reasons for the FEM variations across conditions remain largely unexplored. As mentioned, some studies indicate that FEMs are dependent on the presence of certain endonucleases [65, 138]; however, this is not fully established. A recent study demonstrated that the cfDNA concentration correlated to specific FEM frequencies, indicating that altered cfDNA clearance changes the FEM profile and leads to increased or decreased cfDNA concentrations [68]. We and others have also demonstrated that the underlying gene activity can affect cfDNA FEM frequencies (Fig. 4B,C) [123, 143]. One important concern about research addressing FEMs is the potential biases the chosen NGS methodology introduces. PCR vs. PCR‐free and single‐stranded vs. double‐stranded library preparation methods have the potential to affect FEM proportions [124].

## 5 Cell‐free nucleosomes and histone modifications

Because the lengths of cfDNA fragments represent a ladder that corresponds to the length of DNA wrapped around the histone octamer plus the linker, it is hypothesized that cfDNA is maintained in nucleosomes after they enter into circulation [37, 144, 145]. This is supported by several studies on liquid biopsies showing that the cfDNA concentrations measured with qPCR correlate with the nucleosome concentrations measured with an enzyme‐linked immunosorbent assay (Table 1). As is evident from the correlation coefficients (*r*: 0.29–0.76), the studies have different estimates of how well cfDNA concentrations correlate with circulating nucleosomes. These differences can reflect biological variance or differences between the sample source and/or analysis methods. Nevertheless, at least some of the cfDNA is present in a nucleosome structure. This was proven by the first study on chromatin immunoprecipitation (ChIP) of circulating nucleosomes [146] and later verified in several studies (Table 2).

**Table 1:** Studies evaluating the correlation between cell‐free DNA and nucleosome concentrations.

Source	No. of samples	Correlation coefficient	References
Plasma	92	0.35	Holdenrieder et al. [169]
Serum	92	0.53	Holdenrieder et al. [169]
Plasma	21	0.76	Deligezer et al. [146]
Serum	62	0.29	Wimberger et al. [170]
Serum	111	0.33	Roth et al. [171]

**Table 2:** cfChIP studies on circulating nucleosome PTMs. Most patients had lung, breast, liver, colorectal, or prostate cancer. ddPCR, droplet digital PCR; PTM, post‐translational modification; RCC, renal cell carcinoma; RT‐qPCR, reverse transcription quantitative PCR; WGS, whole‐genome sequencing.

Diseases	Cancer patients	PTMs	Method	References
Myeloma	21	H3K9me1	qPCR	Deligezer et al. [146]
CRC	15	H3K9me3, H4K20me3	FLX‐seq	Gezer et al. [159]
NSCLC	14	H3K36me3	ddPCR	Vad‐Nielsen et al. [160]
Multiple^a^	56	H3K36me3, H3K4me3, H3K4me2, H3K4me1	WGS	Sadeh et al. [35]
NSCLC	5	H3K36me3	ddPCR	Månsson et al. [161]
NSCLC	12	H3K36me3	CAPP‐seq	Trier Maansson et al. [162]
Transplant	0^b^	H3K36me3, H3K4me3, H3K4me2, H3K4me1	WGS	Jang et al. [168]
Multiple^c^	433	H3K4me3, H3K27ac	WGS	Baca et al. [164]
SCLC	82	H3K4me3	WGS	Pongor et al. [167]
NSCLC	12	H3K36me3	CAPP‐seq	Maansson et al. [123]
HCC and CRC	46	H3K4me3, H3K27ac	WGS	Bai et al. [143]
NSCLC	27	H3K4me3, H3K27ac	WGS	El Zarif et al. [165]
RCC	10	H3K4me3, H3K27ac	WGS	El Zarif et al. [163]

a The study includes CRC, several forms of liver diseases, and acute myocardial infarction.

b The study was an investigation of two heart transplant patients.

c The study includes 15 cancers.

### 5.1 Cell‐free chromatin immunoprecipitation

**Fig. 5:**
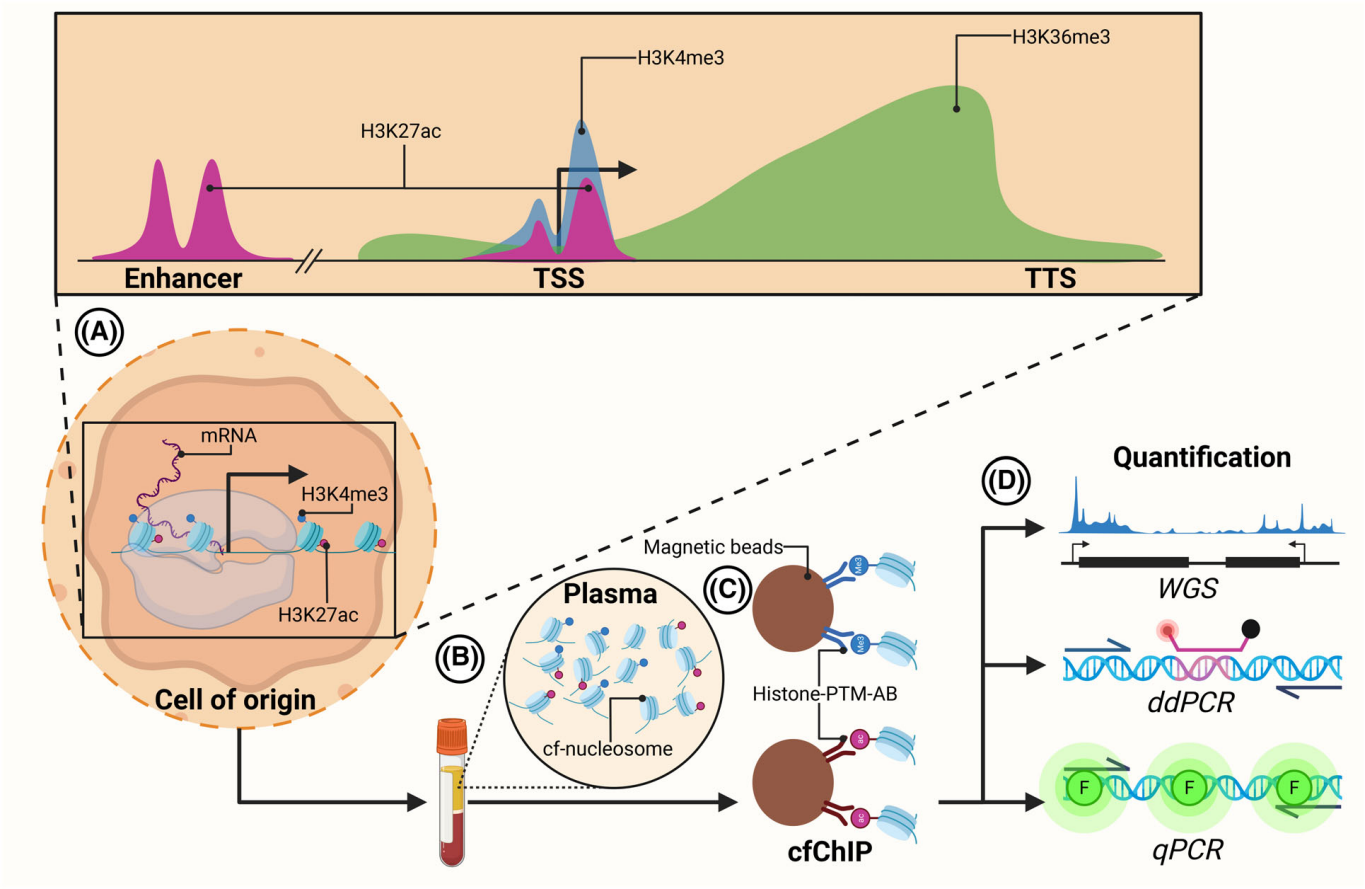
Cell‐free chromatin immunoprecipitation (cfChIP). (A) Messenger RNA (mRNA) expression in the cell of origin is marked by various histone post‐translational modifications (PTMs), such as histone 3 lysine 36 (H3K36me3) or lysine 4 trimethylation (H3K4me3) and histone 3 lysine 27 acetylation (H3K27ac). These modifications have different genomic distributions in actively transcribed genes. H3K27ac (purple) is located in enhancers and at the transcription start site (TSS), H3K4me3 (blue) is around TSS, and H3K36me3 (green) is predominantly in the 3′ end of genes closer to the transcription termination site (TTS). (B) The nucleosomes containing the histone modifications are released into the bloodstream as cell‐free nucleosomes (cf‐nucleosomes). (C) cfChIP isolates specific cf‐nucleosomes using magnetic beads coupled to antibodies specific for different histone‐PTMs (histone‐PTM‐AB). (D) The cfChIP enrichment is then quantified with whole‐genome sequencing (WGS), droplet digital PCR (ddPCR), or quantitative PCR (qPCR) with a fluorescent reporter (F).

Briefly speaking, PTMs are present on the histones in the nucleosome, and the nature of the PTMs reflects the epigenetics and hence the transcriptional activity of the underlying gene or chromosomal region (Fig. 5A). PTMs are conserved in circulating nucleosomes (Fig. 5B), as shown by Van den Ackerveken et al. [147], who used a proteomic approach to quantify the levels of multiple histone modifications in plasma from healthy individuals and CRC patients. Moreover, the DNA associated with the circulating nucleosomes can be analyzed with cell‐free chromatin immunoprecipitation (cfChIP) to infer the transcriptional activity of the corresponding genomic region in the cell of origin. With cfChIP, nucleosomes containing specific PTMs are directly isolated from plasma using histone‐PTM antibodies (Fig. 5C), and cfDNA enrichment is quantified using either a targeted approach with, for example, ddPCR or genome‐wide with WGS (Fig. 5D).

#### 5.1.1 Histone modifications and gene transcription

Several studies have been published using cfChIP (Table 2). The studies have addressed various types of PTMs, and in the following section, the characteristics of the most studied PTMs are described before we continue to the cfChIP studies on liquid biopsies.

Histone 3 lysine 36 trimethylation (H3K36me3) is associated with the 3′ end of actively transcribed genes [148]. The methylation of lysine 36 is mediated by SETD2, which also serves as a tumor suppressor given its involvement in transcription regulation and DNA repair [149]. H3K36me3 located in the gene body promotes chromatin condensation, which prevents spurious transcription from cryptic start sites within the gene body [150]. Importantly, recent findings illustrate that the level of H3K36me3 in a given gene correlates with the amount of RNA transcribed from the gene [151]. Another strong signal of transcription is histone 3 lysine 4 trimethylation (H3K4me3), which is located in the active promoters [152]. H3K4me3 is one of the most well‐studied histone PTMs, and levels of H3K4me3 correlate with RNA expression levels [153]. The H3K4me3 peak at active promoters is narrow compared with that of H3K36me3, which exists throughout the gene body [154]. Multiple methyltransferases are responsible for the trimethylation of lysine 4, where SETD1A and SETD1B are thought to be the primary transferases in mammalian cells. The role of H3K4me3 is complex but is thought to be a result of transcription initiation and not the causal effect leading to transcription [152]. However, it has been determined that H3K4me3 plays an important role in transcriptional pause‐release and hence the first steps of transcription elongation [155]. Acetylation of histone lysine residues is also a common PTM, and H3K27ac correlates with H3K4me3 levels in active promoters [156]. H3K27ac is also located in enhancers, where its role is to distinguish active enhancers from inactive or poised enhancers containing H3K4me1 alone [157]. Therefore, H3K27ac is widely used to identify active enhancers [158].

### 5.2 cfChIP studies in cancer research

The first two cfChIP studies focused on repetitive genomic elements [146, 159]. The field has since turned to studies of histone epigenetics at the gene‐specific level, particularly the PTMs H3K4me3, H3K27ac, and H3K36me3. Vad‐Nielsen et al. [160] demonstrated that H3K36me3 cfChIP enrichment followed by ddPCR of *KRT6ABC*, *ACTG1*, and *SAT2* could be used to distinguish LUSC from LUAD patients. Later, tumor‐specific expression was detected by quantifying the *EGFR‐L858R* allele fraction with ddPCR in H3K36me3 cfChIP samples from NSCLC patients [161]. Comparing the cfChIP enrichment of the *EGFR‐L858R* mutation with *EGFR‐WT* revealed a greater enrichment of the mutant allele corresponding to high gene expression of *EGFR‐L858R* in the NSCLC tumor. Using ddPCR for cfChIP quantification allows for the estimation of the gene expression of known driver mutations, such as *EGFR*. However, ddPCR limits the expression profile to a few genes or alleles. In addition, random variation between samples in the genomic distribution of histone modifications can affect cfChIP‐ddPCR enrichments. Because ddPCR amplicons are limited to approximately 100 bp, only a fraction of the region with the histone modification is used for quantification, which can induce biased enrichment estimates. By utilizing targeted sequencing to quantify cfChIP enrichment [162], genomic coverage is increased, yielding a more comprehensive profile of the histone‐PTM distribution. In addition, the high sequencing depths allow allele‐specific enrichments to be quantified to detect tumor‐specific gene expression. However, targeted sequencing is also limited by the number of genes and the capture regions of the panel compared with WGS. For example, H3K4me3 and H3K36me3 are differently distributed relative to the TSS (Fig. 5A), meaning custom gene panels have to be designed for individual histone modifications. Nevertheless, targeted sequencing was used to detect gene expression differences between NSCLC and SCLC patients with H3K36me3 cfChIP [162]. Here, the relative gene enrichment correlated with RNA expression patterns estimated from NSCLC and SCLC cell lines. Moreover, NSCLC patients had higher enrichment of, for example, *EGFR*, whereas SCLC patients had higher cfChIP enrichment of *KIF19* and *CRMP1*, which are highly expressed in SCLC tumors.

Sadeh et al. [35] published a cfChIP WGS (cfChIP‐seq) study with various types of patient samples and targeted multiple histone modifications in their assays. They demonstrated how genome‐wide H3K4me3 enrichment can be used to determine the cfDNA TOO, which reflects the disease location in the patients. For example, they demonstrated how liver cfDNA dynamics measured with cfChIP‐seq correspond to circulating alanine aminotransferase levels in hepatectomy patients. They also identified significantly enriched genes in CRC patients and used this to distinguish CRC patients from healthy individuals and to identify several CRC subtypes enriched for different molecular pathways. They were the first to apply WGS for cfChIP quantification, showing great utility for multiple histone modifications. Although the sequencing depths are low, making enrichment of somatic mutations unviable, the genome‐wide approach enables large molecular gene networks to be identified. In addition, WGS simplifies the workflow and does not require any prior knowledge regarding the distribution of the histone modification analyzed with cfChIP. Similarly, in another study, gene expression differences between renal cell carcinoma (RCC) subtypes were evaluated with H3K27ac cfChIP‐seq and cfDNA methylation profiling [163]. They revealed a high enrichment of *FOSL1* in sarcomatoid RCC compared with epithelioid RCC patients. Epithelioid RCC patients treated with the tyrosine kinase inhibitor, sunitinib, with high *FOSL1* cfChIP enrichment had a worse outcome than epithelioid RCC patients with low *FOSL1* cfChIP enrichment. The epithelioid RCC patients had a better response to sunitinib than sarcomatoid RCC patients. However, the sunitinib response in epithelioid patients with high *FOSL1* cfChIP enrichment was similar to that of sarcomatoid RCC patients. This illustrates how cfChIP‐seq can be used to identify patients with a reduced response to specific treatment strategies based on the enrichment in key genes independently of the histological subtype. Baca et al. [164] applied H3K27ac and H3K4me3 cfChIP‐seq to 15 types of cancer and demonstrated that the information acquired from both H3K27ac and H3K4me3 cfChIP can be used to identify active genes. They found a strong positive correlation (*r* = 0.95) between the plasma H3K4me3 cfChIP signal in healthy individuals and whole‐blood RNA expression. However, only a modest negative correlation was observed between cfMeDIP‐seq enrichment and whole‐blood RNA expression (*r* = −0.16). These results suggest that cfChIP‐seq could be superior to methylation profiling for single‐gene inferred RNA expression. In addition, by correlating the cfChIP‐seq gene enrichment signal for both H3K4me3 and H3K27ac modifications to the plasma tumor fraction estimated with ichorCNA, they identified cancer‐specific genomic regions classified as correlated regulatory elements. Using these regions, they differentiated between cancer patients and healthy individuals and classified tumor gene expression programs.

Analyzing serial blood samples taken longitudinally from EGFR‐positive NSCLC patients, El Zarif et al. [165] detected NSCLC‐to‐SCLC transformation with H3K4me3 and H3K27ac cfChIP‐seq, whole‐genome methylation profiling with cfMeDIP‐seq, and nucleosome footprints inferred by WGS. For each feature, they quantified an SCLC risk score and showed that each one can differentiate between LUAD and SCLC transformation patients, with an AUC between 0.70 and 0.87. The H3K27ac cfChIP‐seq‐ and cfMeDIP‐seq‐based classifiers had similar performance, with AUCs of 0.87 and 0.85, respectively. In contrast, the H3K4me3 cfChIP‐seq‐based classifier only had an AUC of 0.70, with a strong overlap in SCLC risk score between NSCLC and SCLC‐transformed patients. Importantly, the SCLC‐enriched sites from nucleosome footprints, methylation, and H3K27ac cfChIP‐seq were nonoverlapping, illustrating how each epigenomic feature complemented the others. Based on these features, they created an integrated epigenomic cfDNA classifier with an AUC of 0.94, demonstrating that a multimodal classifier results in improved diagnostic accuracy. The H3K4me3 cfChIP‐seq signal was excluded from the integrated classifier based on poor performance, highlighting the importance of feature selection in epigenomic ML models.

In 2022, Fedyuk et al. [166] developed the epigenetics of plasma‐isolated nucleosomes (EPINUC), which is an image‐based approach to quantify circulating protein and epigenetic biomarkers. EPINUC utilizes biotinylated antibodies bound to a streptavidin surface to quantify several histone modifications, including H3K4me3, H3K27me3, and H3K36me3, and circulating proteins, such as CEA and TP53. Moreover, EPINUC‐seq adds poly(A) tails to circulating nucleosomes and captures them on a PEGylated‐poly(T) surface whereby fluorescently labeled antibodies can identify the histone modifications. Next, single‐molecule sequencing reveals the DNA associated with the nucleosomes, enabling sequences to be integrated with the histone‐PTM profiles. The authors demonstrated how EPINUC‐seq establishes differential H3K4me3 and H3K27me3 epigenetic profiles between cancer patients and healthy individuals. For example, EPINUC‐seq H3K27me3 profiles in a CRC patient with liver metastasis overlapped with colon and liver H3K27me3 ChIP‐seq peaks, and EPINUC‐seq H3K4me3 signals were concordant with CRC H3K4me3 cfChIP‐seq peaks Sadeh et al. [35] identified. Both cfChIP‐seq and EPINUC‐seq create epigenetic profiles based on PTMs of circulating histones although the strategies are markedly different. Although EPINUC‐seq enables the identification of nucleosome PTMs and other circulating proteins, the simpler workflow and potential higher throughput of cfChIP‐seq could be beneficial for the detection of tumor‐associated nucleosomes.

The study of nucleosome PTMs in plasma has also assisted other aspects of liquid biopsies. Pongor et al. [167] verified the discovery of extrachromosomal circular DNA (eccDNA) containing the *MYC* oncogene with H3K4me3 cfChIP‐seq. Patients with eccDNA containing *MYC* had high *MYC* cfChIP enrichment and high enrichment in genes affected by high MYC activity. Fragmentomic features of cfDNA originating from genes with high activity have been explored in several cfChIP‐seq studies. Recently, two studies used cfChIP‐seq of H3K36me3, H3K4me3, or H3K27ac to identify which genes were active in the cell of origin and identified cfDNA fragmentomic features enriched for these genes [123, 143]. These results are highly interesting and can be used to infer the nucleosome PTM profile for a given region in the cell of origin based on cfDNA fragmentomic features alone, such as fragment lengths, FEMs, and nucleosome footprints. Bai et al. deduced H3K27ac and H3K4me3 signals from cfDNA fragmentomes to infer the cfDNA tissue of origin in healthy individuals. In addition, they identified HCC patients and CRC patients with liver metastasis based on cell‐free deduced liver‐specific H3K27ac signals [143].

## 6 Conclusion and perspectives

The field of cfDNA epigenetics is rapidly expanding and is being studied in multiple clinical settings. The studies on nucleosome PTMs with cfChIP have been under rapid development in the last half‐decade and have expanded to WGS studies classifying genome‐wide enrichment. The method has significant potential; however, its clinical utility remains largely unexplored and few studies have been conducted to compare cfChIP results in serial blood samples for patients treated with a specific therapy. Jang et al. [168] demonstrated that cfChIP is also applicable beyond cancer and used cfChIP‐seq to identify heart tissue damage in heart transplant patients.

cfDNA methylomics has been explored in more studies over many years. However, the recent development of Duet sequencing and the expansion of native cfDNA sequencing with the nanopore technology will likely broaden the field to focus on genome‐wide 5mC and 5hmC signals. This approach will also allow fragmentomic features to be investigated alongside cfDNA methylation profiles. This is crucial, given that multiple studies have demonstrated how the integration of several epigenetic features in multimodal ML classifiers outperforms algorithms based on single features [124, 143, 165]. Most proof‐of‐concept studies on liquid biopsy epigenetics have been conducted on late‐stage cancer patients to, for example, detect collateral damage of distant metastases [35, 143, 166]. Using multimodal ML classifiers based on several epigenomic features, for example, 5mC/5hmC, fragmentomics, and histone modifications, could increase the sensitivity of these strategies [165], which could enable epigenetic approaches to be implemented in the early detection of cancer. cfDNA epigenetics also provide more integrated knowledge about the tumor cells than mutation‐based ctDNA techniques. However, the molecular subtypes or large‐scale gene networks identified in the individual studies lack clinical validation. Understanding these results' applicability in a clinical context must be the focus of future research to move the field of cfDNA epigenetics closer to impacting the outcome for cancer patients.

## Conflict of interest

The authors declare no conflict of interest.

## Author contributions

All authors contributed to the conception and design of the manuscript. CTM wrote the body of the manuscript. CTM, ALN, and BSS performed manuscript revision and editing. CTM created the artwork. All authors read and approved the final manuscript.
